# Tailoring and field-testing the use of a knowledge translation peer support shared decision making strategy with First Nations, Inuit and Métis people making decisions about their cancer care: a study protocol

**DOI:** 10.1186/s40900-018-0085-3

**Published:** 2018-03-01

**Authors:** Janet Jull, Maegan Mazereeuw, Amanada Sheppard, Alethea Kewayosh, Richard Steiner, Ian D. Graham

**Affiliations:** 1 Ottawa Hospital Research Institute and University of Ottawa, Ottawa, Ontario Canada; 2 Aboriginal Cancer Control Unit, Cancer Care Ontario, Toronto, Ontario Canada

**Keywords:** First Nations, Inuit and Métis people, Shared decision making, Decision coaching, Peer support, Integrated knowledge translation, Cancer care

## Abstract

**Plain English summary:**

Tailoring and testing a peer support decision making strategy with First Nations, Inuit and Métis people making decisions about their cancer care: A study protocol.

First Nations, Inuit and Métis (FNIM) people face higher risks for cancer compared to non-FNIM populations. They also face cultural barriers to health service use. Within non-FNIM populations an approach to health decision making, called shared decision making (SDM), has been found to improve the participation of people in their healthcare. Peer support with SDM further improves these benefits. The purpose of this study is to tailor and test a peer support SDM strategy with community support workers to increase FNIM people’s participation in their cancer care.

This project has two phases that will be designed and conducted with a Steering Committee that includes members of the FNIM and cancer care communities. First, a peer support SDM strategy will be tailored to meet the needs of cancer system users who are receiving care in urban settings, and training in the SDM strategy developed for community support workers. Three communities will be supported for participation in the study and community support workers who are peers from each community will be trained to use the SDM strategy.

Next, each community support worker will work with a community member who has a diagnosis of cancer or who has supported a family member with cancer. Each community support worker and community member pair will use the SDM strategy. The participation and experience of the community support worker and community member will be evaluated.

The research will be used to develop strategies to support people who are making decisions about their health.

**Abstract:**

Tailoring and field-testing the use of a knowledge translation peer support shared decision making strategy with First Nations, Inuit and Métis people making decisions about their cancer care: A study protocol

**Background**

First Nations, Inuit and Métis (“FNIM”) people face increased cancer risks in relation to general populations and experience barriers to health service use. Shared decision making (SDM) has been found to improve peoples’ participation and outcomes in healthcare and peer support with SDM further improves these benefits. The purpose of this study is to tailor and then field test, by and with FNIM communities, a peer support SDM strategy for use in cancer care.

**Methods**

This project has 2 theory-driven phases and 5 stages (a-e). A core research team that includes members of the Aboriginal Cancer Control Unit of Cancer Care Ontario communities and academic researchers, will work with a Steering Committee. In **phase 1**, (stage a) a peer support SDM strategy will be tailored to meet the needs of cancer system users who are receiving care in urban settings and (stage b), training developed that will i) introduce participant communities to SDM, and ii) train community support workers (CSWs) within these communities. Next (stage c), three communities will be approached for voluntary participation in the study. These communities will be introduced to SDM in community meetings, and if in agreement then CSWs from each community will be recruited to participate in the study. One volunteer CSW from each community will be trained to use the peer support SDM strategy to enable phase 2 (field test of the peer support SDM strategy).

During **phase 2** (stage d), each CSW will be matched to a volunteer community member who has had a diagnosis of cancer or has supported a family member with cancer and is familiar with Ontario cancer systems. Each CSW-community member pair (3 to 4 pairs/community) will use the tailored peer support SDM strategy; their interaction will be audio-recorded and their participation and experience evaluated (total of 9 to 12 interviews). As well (stage e), data will be collected on health systems’ factors related to the use of the peer support SDM strategy.

**Discussion**

Findings will develop peer support SDM strategies to enhance participation of FNIM people in cancer care decisions, advance knowledge translation science, and support a proposal to conduct a multi-site implementation trial.

## Background

First Nations, Inuit and Métis (“FNIM”) populations who are living in Canada have disproportionate and increasing rates of cancer relative to the general population. For First Nations compared to the general population, cancer incidence is rising more rapidly (especially colorectal cancer) [1] and survival is poorer for several cancer types, including the four most common: breast, prostate, colorectal and lung [2]. Although little is known about cancer in Ontario’s Inuit and Métis populations,^1^ evidence is accumulating that their cancer rates are disproportionately higher, and in particular for lung cancer, compared to non-FNIM populations [3, 4].

Western healthcare models reflect values, knowledge systems, and care practices that may not align with those of FNIM people [5, 6] and with impacts on the participation of FNIM people in mainstream healthcare settings [5, 7–10]. There is a long history of colonization and loss of cultural identity which continue to have dramatic impacts on ways of life and all aspects of health. While FNIM people demonstrate tremendous cultural resilience [11], FNIM people experience ongoing marginalization and poor health and well-being as a result of complex colonial relations of power [12, 13] within Canadian society and that include health systems [5]. As well, misunderstandings can exist between health providers and FNIM clients and may affect the ability of health providers to help their clients achieve optimal health. Health providers may not understand the perspectives of an FNIM client: when they cannot clearly and meaningfully communicate with an FNIM client, they may treat the client as culpable for misunderstandings or being disinterested in their healthcare. For example, the higher (in relation to general populations) cancer mortality among Aboriginal people in Australia is attributed to poor detection and screening access, as well as inadequate and culturally inappropriate treatment and support services [10]. Western-trained healthcare providers typically lack understandings of diverse FNIM cultures [6, 14] and this has had a negative impact on the health of FNIM people [15], as well as affecting their participation in healthcare settings, including cancer care [10, 16]. These factors can ultimately result in poor health status, marginalization within the health system and increase risk for experiences of racism for FNIM people [17, 18].

Shared decision making (SDM) is an important evidence-informed strategy that holds the potential to promote peoples’ participation in their health decisions, and could contribute to better health system participation and improved healthcare outcomes for FNIM people. SDM is a central feature of client-centred care [19, 20]. It engages healthcare providers with people in health decisions [21], increases participation in health settings and fosters knowledge translation (KT) [22] in a process of information sharing between healthcare providers and users. SDM is enacted through healthcare delivery approaches and tools [23, 24]. As well as structuring a collaborative, client-centred approach between practitioner and client, SDM tools and approaches promote the sharing and use of information on the benefits and harms of care options. Further, SDM has been found to increase people’s involvement in making more informed and values-based care decisions [24, 25] and to be of particular benefit to populations that experience health and social disadvantage (i.e., health equity gaps) [26]. SDM approaches and tools are currently being developed and implemented in international settings and are evolving as standards of care [19, 20]. SDM has considerable potential to narrow health equity gaps by engaging FNIM people with their healthcare providers in decision making. Interventions to engage FNIM populations collaboratively in making decisions about their health are under development [27] and the work proposed here contributes to the development of the evidence base that may be used to design a future intervention study to measure the effectiveness of a SDM intervention. SDM has begun to be explored with these populations along with peer support which has been identified as an important feature of this work [28, 29].

Peer support is defined by Dennis as “the provision of emotional, appraisal, and informational assistance by a social network member who possesses experiential knowledge of a specific behaviour or stressor and similar characteristics as the particular population” [30]. In short, peer support workers extend health systems reach [31]. Within Ontario’s cancer systems community-based peers provide community-based health systems peer support to First Nation, Inuit and Métis communities. These community peers are referred to by different names such as Community Health Representatives, Home Care Workers, or Personal Support Workers. Collectively, FNIM community-based peers can be called “community support workers” (“CSWs”). Ensuring health equity for all Ontarians across the cancer system is one of the six goals identified by Cancer Care Ontario in the fourth Ontario Cancer Plan (OCP IV) [32]. Within this goal, Ontario’s FNIM people are accorded a priority focus. The OCP IV commits Cancer Care Ontario to address the needs of FNIM people through the implementation of the third Aboriginal Cancer Strategy (ACS III), the vision of which is “to improve the performance of the cancer system with and for First Nations, Inuit and Métis peoples in a way that honours the Aboriginal Path of Well-being” (p. 7) [33] and that is led by members of Cancer Care Ontario’s Aboriginal Cancer Control Unit. Cancer Care Ontario’s Aboriginal Cancer Control Unit networks and resources are well-positioned to utilize peer support SDM through engagement of CSWs to enhance participation of FNIM populations making decisions about their cancer care.

### Research approach

Given the health and social systems and structures that undermine the health and well being of FNIM populations, it is imperative that research to explore and adapt current approaches to SDM be conducted in full collaboration with FNIM people [34]. Prior to development of this proposal, a series of studies was conducted in full-partnership with an urban-based FNIM community, and a process of adaptation and usability testing resulted in the development of the peer-support SDM strategy [27–29, 35]. The work was conducted from within a mutually agreed upon partnership and ethical framework, and used a process aligned with the socio-cultural values of those in the partnership [36].

Health research is conducted with the expectation that it advances knowledge and eventually translates into improved health systems and health outcomes. The research study proposed here will be conducted in full partnership with cancer care health system and community partners (“knowledge users”). The partnership will be guided by a mutually agreed upon ethical framework (e.g., Ownership, Control, Access and Possession™ [37]) and an agreed upon theoretical approach such as postcolonial theory [38], that provides a theoretical lens to show everyday experiences of marginalization of FNIM people that occur in systems structuring human relations such as healthcare settings [39, 40]. Postcolonial theory may be used to implement research processes that examine SDM while promoting a decolonizing agenda [38]. Community-based participatory methods will be used as an appropriate approach for engaging in a research partnership that fosters integrated knowledge translation.

Knowledge Translation (KT) is defined by the Canadian Institutes of Health Research (CIHR) [41] as a dynamic and iterative process that includes synthesis, dissemination, exchange and ethically sound application of knowledge to improve health, provide more effective health services, products and systems. Integrated KT is an approach to research that involves engaging knowledge users (those who can act on research findings) in the research process from defining the research question to application of findings [42]. Integrated KT research is undertaken with the expectation that the outputs of the research will be culturally relevant, useful, and useable, and more likely to be applied in practice and policy and create greater capacity to use research by knowledge users. For these reasons, an integrated KT research approach is therefore also expected to produce more timely impacts. The proposed research adopts an integrated KT approach and was initiated following engagement with members of Cancer Care Ontario’s Aboriginal Cancer Control Unit, and Cancer Care Ontario’s Joint Ontario Aboriginal Cancer Committee (https://www.cancercareontario.ca/en/cancer-care-ontario/programs/aboriginal-programs/joint-cancer-care-aboriginal-committee). The Joint Ontario Aboriginal Cancer Committee is comprised of leadership from each of the First Nation Provincial Territorial Organization’s and independent communities, the Métis Nation of Ontario and Tungasuvvingat Inuit. The committee provides essential input and guidance on all the work that is performed by the Aboriginal Cancer Control Unit including reviewing this strategy at their latest gathering.

**Fig. 1:**
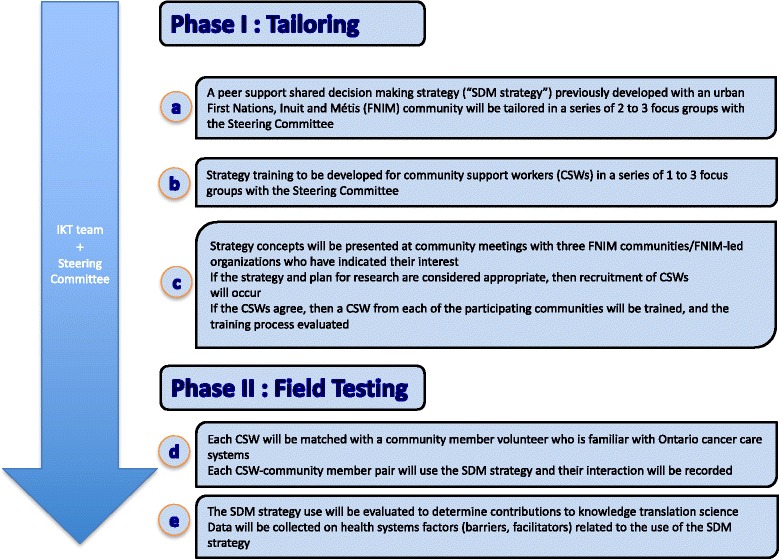
The two-phase, five-stage study

The processes integral to integrated KT build the opportunity for researchers and knowledge users to work collaboratively and utilize the expertise that each brings to the partnership [43] (Fig. 1). A previously developed and used collaborative approach to community-research partnerships [36] shall be utilized to foster an integrated KT approach.

The proposed research study has been developed in complete collaboration between academic researchers and members of the knowledge user organization that make up the Integrated KT team (“IKT team”), who are decision makers in the research study and co-authors of this proposal (JJ, MM, AS, AK, RS, IG). A previously developed peer support SDM strategy developed by and with an urban-based FNIM community [28] in Ontario will be tailored and then field-tested in urban settings for its use and potential to enhance participation of FNIM people making cancer treatment decisions.

### Objective and research questions

The objective of the proposed two-phase, five-stage study is to tailor and field-test the use of a knowledge translation peer support SDM strategy to enhance participation of FNIM people in decisions about their cancer care and will also build evidence about KT science (Fig. 1). The study will answer the following research questions:

1. How might a previously developed peer support SDM strategy need to be tailored to enhance participation of FNIM people in making decisions about their cancer care?
2. What training is needed to deliver the strategy to CSWs?
3. Do the CSWs learn how to use the peer support SDM strategy in the training program?
4. How acceptable, useful/relevant is the training perceived by trainee CSWs?
5. Does the peer support SDM strategy support elements of SDM?
6. Do CSWs and community members perceive the peer support SDM strategy as acceptable, useful, and relevant?
7. What is the perceived feasibility of using the tailored peer support SDM strategy within cancer care systems?
8. How can integrated KT research be conducted with FNIM communities?
9. What are the perceived determinants (that is barriers and facilitators) for implementation of the peer supported SDM strategy?

## Methods

The IKT team will be involved in and provide leadership throughout the 2 phases of the study that tailor (phase 1) and then field-test (phase 2) a peer support SDM strategy (“SDM strategy”) (Fig. 1). The IKT team will recruit and work closely throughout the study with a Steering Committee consisting of members that span the Ontario cancer system and who are familiar with the settings where cancer care delivery occurs for those likely to participate in this study (that is, urban settings of care). As the Cancer Care Ontario Aboriginal Navigators are linked closely to CSWs and FNIM communities in Ontario, the progress of the study will be reported at their monthly meetings for their monitoring and/or input. The two study phases are described across 5-stages (a-e): Phase 1 includes a) tailor the SDM strategy, b) develop training for CSWs in the use of the SDM strategy, c) train CSWs in the use of the SDM strategy, and Phase 2 includes d) field test the SDM strategy, e) evaluate the use of the SDM strategy to determine contributions to the science of KT.

The Ottawa Health Science Network Research Ethics Board (OHSN-REB) granted ethics approval for this study in May 2017 (#20170150-01H).

### Phase 1

Tailor a knowledge translation strategy to enhance participation of FNIM people in decisions about their cancer care.

#### Research question 1 (stage a)

How might a previously developed peer support SDM strategy need to be tailored to enhance participation of FNIM people in making decisions about their cancer care?

*Design*

A Steering Committee will tailor a previously developed peer support SDM strategy for use by CSWs, who support people that make cancer care decisions. Consensus building methods, that is, a structured process of deliberation and debate about concepts among members, will be used with the Steering Committee to iteratively tailor the SDM strategy (or strategies, to meet the preferences/needs of different FNIM-user community groups) with documentation of decision points in the process. The resulting SDM strategy will be agreed upon by the IKT team.

*Intervention for adaptation*

The SDM strategy consists of the Adapted Ottawa Personal Decision Guide used with decision coaching and is designed to foster SDM [28]. It is based on the Ottawa Personal Decision Guide (OPDG), a tool that is validated for use in making any health-related or social decision. It can be used to help people summarize their current knowledge (of options, benefits, and harms), clarify their values associated with option outcomes, plan the next steps, and track their progress in decision making. The OPDG underwent a process of adaptation by and with the community using focus groups and usability testing, to become the Adapted OPDG, and for which FNIM participants recommended the use of a decision coach, and that is reported in detail elsewhere [28, 35]. Decision coaching is non-directive decision support provided by a trained individual. Training in the use of the Adapted OPDG with decision coaching has been developed as a workshop [44].

*Participants and procedures*

Participants will be Steering Committee members who live in Ontario and who are members of the Cancer Care Ontario network of the Aboriginal Cancer Control Unit. The invitation to join the 7 to 9 member Steering Committee will be extended to individuals who themselves are members of FNIM communities and/or work with FNIM people who are in Ontario cancer care systems. The Steering Committee will consist of representatives of the Patient Family Steering Committee, CSWs, community health directors, members of the First Nations-Inuit-Métis Regional Health Tables, and healthcare providers. An important first step in the Steering Committee members’ work together involves the development of terms of reference, and in which Steering Committee members can indicate their preferences for when and how to meet as well as on points such as facilitation of the group meetings. It is proposed that they will be asked to participate in 2 to 3 focus groups conducted on-line, in an iterative process of tailoring the strategy to meet the needs of FNIM people accessing care situated in an urban setting and who are interacting with Ontario’s cancer system. The focus groups are proposed to be facilitated by the researcher(s) and a research assistant. They are anticipated to take up to 1 h each. Although the Steering Committee is to be considered a partner in the research, informed consent from members will be sought as information will be gathered from the Steering Committee during the study processes. Non-identifying demographic information will be collected from Steering Committee members, and they will then be guided as a group by the researcher(s) through use of the strategy with an example decision. The Steering Committee members will be asked to provide general feedback (for instance, organization, readability of the decision guide). Then, the strategy will be reviewed step by step to discuss specific adaptations to language and/or flow of ideas, and to document a rationale for changes. Field notes and/or audio recording by the researcher(s) and/or the research assistant will document the input by the Steering Committee members.

*Analysis*

Data will be organized to map the adaptation process and summaries about the SDM strategy tailoring will be developed and analyzed for criteria indicating equivalence between the original and end product(s). The findings will be reflected back to the Steering Committee for further input, and then to the IKT team and any additional information will be incorporated into the tailored strategy(ies).

#### Research question 2 (stage b)

What training is needed to deliver the strategy to community support workers?

*Design*

The Steering Committee will be engaged in consensus building methods during an iterative process of focus groups to develop both strategy training objectives and processes. The training development will be documented during the focus groups, and in consultation with the IKT team until there is full agreement on both strategy training objectives and process among IKT team members.

*Participants and procedures*

Participants will be the Steering Committee members who will be invited to participate in 1 to 3 focus groups in an on-line process of developing training for the strategy. The focus groups are anticipated to take up to 1 h each and to be facilitated by researcher(s) and a research assistant. The Steering Committee will be guided as a group by the researcher through SDM strategy training using a previously developed SDM workshop based on the original strategy. The group will be asked to provide feedback on the training objectives and processes, and field notes and/or audio recording by the researcher and/or the research assistant will document Steering Committee input.

*Analysis*

Data will be organized to map the training development process and summaries developed to explain the development of the end product(s). The final training product(s) will undergo a pilot test within the IKT team to ensure no further adaptations are required, and will then be agreed upon by the IKT team members and the Steering Committee.

#### Research questions 3,4 (stage c)

Do the CSWs learn how to use the peer support SDM strategy in the training program?

How acceptable, useful/relevant is the training perceived by trainee CSWs?

*Design*

Consensus building methods will be used to introduce and seek approval from urban-based FNIM communities and the CSWs for the use of the strategy.

*Participants and procedures*

Steering Committee members will identify potential community partners to be invited to learn about the study and will be communities situated in urban settings. Members of the IKT team will make contact with the communities and inform them of the study; if interested, a community agreement can be used to structure conversation about possible engagement in the research study. If needed, adjustments to the proposed research strategy can be made. Then, a plan to introduce the study to the community will be enacted. Participants will be general FNIM community members and will include CSWs. Community members will be purposefully invited (using posters, email) to participate with the researcher(s) in an English or Inuktitut^2^ introductory presentation about the SDM strategy and the research plan. If the community agrees with the proposed study aims, then a local CSW will be purposefully invited to participate in the strategy training following the community meeting.

It is proposed that the SDM strategy will be introduced at a community meeting by the researcher(s) and a research assistant to 3 FNIM communities who have responded to invitations to learn about the research study and the SDM strategy. The proposed method of introductions to community are subject to modification depending on preferences of community for necessary research approvals. Response to the community presentation about the SDM strategy will be documented in field notes by the researcher(s). If needed, adaptations to the SDM strategy/presentation will be made to ensure relevance to the particular community/ies. The appropriateness of the SDM strategy concepts will be indicated by community leaders through the granting of permission to recruit study participants (i.e., no objection to recruitment of community CSWs and community members).

If the community indicates that the SDM strategy and plan for research are appropriate, then the CSW from 3 communities will be invited to participate in the study. After reviewing and signing the consent form with the researcher, the CSW will be asked for some basic demographic information and then will be trained in the SDM strategy and their experience evaluated. CSW agreement to participate and training attendance will be considered to indicate SDM strategy concept appropriateness. Training acceptability, usefulness and relevance will be indicated by CSW training attendance and demonstration of learning objectives, and CSW self-report.

*Analysis (community meetings)*

Expressions of concern or acceptance from the communities documented in field notes by the researcher will be entered into an Excel database and analyzed descriptively by the researcher, reviewed with the IKT team and with the Steering committee for input and approval of findings.

*Analysis (CSW training)*

Information on acceptability, usefulness and relevance of the training will be gathered through (written) documented observations in field notes, and in the CSW interviews that are conducted post-training. Researcher field notes will be entered into an Excel database and analyzed descriptively by the researcher. Adjustments to the strategy training objectives or process will be mapped to decision points. Post-training interviews will be analyzed to seek demonstration for evidence of self-efficacy and learning objectives, that is, for evidence that demonstrates (such as in role play, during discussion) how to use SDM in their practice. Findings will be reviewed with the IKT team and Steering Committee.

### Phase 2

Field-test the use of a knowledge translation SDM strategy to enhance participation of FNIM people in decisions about their cancer care and will also build evidence about KT science.

#### Research questions 5,6,7 (stage d)

Does the peer support SDM strategy support elements of shared decision making?

Do CSWs and community members perceive the peer support SDM strategy as acceptable, useful, and relevant?

What is the perceived feasibility of using the tailored peer support SDM strategy within cancer care systems?

*Design*

A field test of the strategy will be conducted and the interaction between CSWs and matched community members evaluated. The SDM strategy will be evaluated for evocation of elements of SDM and to understand whether and how the CSWs and community members perceive the SDM strategy to be acceptable, useful and relevant, and the feasibility of its use. Our interpretation of success will be that of gaining consensus among community partners about the content and method of delivery of the intervention. As well, it is anticipated that the Steering committee and IKT team will define success based on criteria that are appropriate to the community and reflect the use of the SDM strategy: for example, do CSWs and community members feel satisfied with the interaction, does it help them to feel informed, do they indicate feeling supported, do they think it will help to foster better participation in making health decisions with their healthcare providers.

*Participants and procedures*

Community members who have had a diagnosis of cancer or has supported a family member with cancer and is familiar with Ontario cancer systems from each community will be recruited for participation using posters as a handout or posting of them by CSWs and according to community protocol. Following a process of informed consent, community members will be asked some basic demographic information.

The use of the SDM strategy will be evaluated in a 30 to 60-min role-play of preparation to make a decision with a healthcare provider. The CSW and community member will choose a decision that is preferred by the community member before the role-play. The interaction between a CSW and 3 to 4 community members will be recorded and/or observed by the researcher (a total of 3 to 4 interactions per community). A standardized tool [45] and thematic analysis of the recorded and/or observed interactions will be used to evaluate the CSW-community member interaction for elements of SDM. Pre- and post-interaction surveys (for acceptability) and recorded and/or observed post interaction semi-structured interviews (usefulness, relevance) with the researcher will be used to evaluate CSW and community member experience.

To understand the feasibility of the strategy use, data will be collected on health systems’ factors that reflect barriers and facilitators and that are from researcher reflective notes and post-interaction interviews with CSW and community members. In addition, community health managers will be invited to comment on the feasibility of the SDM strategy. Following a process of informed consent, the manager will be asked for demographic information and then to indicate their preference to either participate in a short survey and/or interview related to the perceived feasibility, that is, barriers and facilitators in their area of the health system, for the use of the SDM strategy.

The Steering Committee and IKT team will agree (in consultation with respective communities) upon key criteria to determine the indicators for SDM strategy evaluation, that is, to identify whether the SDM strategy is favourable or unfavourable to evoke SDM, as well as the SDM strategy acceptability, usefulness, relevance and feasibility (see analysis).

*Analysis (evidence for elements of shared decision making; perceived to be acceptable, useful, and relevant)*

The recorded and/or observed interaction between CSW-community member pairs will be analyzed using a standardized tool and a six-step thematic analysis [40] of the interaction to describe elements of SDM. Pre- and post-interaction surveys (for acceptability of SDM strategy) and post interaction semi-structured interviews (usefulness, relevance of SDM strategy) of CSW and community member experiences will be recorded and evaluated using thematic analysis to identify content (for evidence of strategy concepts) and process (for evidence of SDM strategy delivery approach) factors and contextualized using a postcolonial theoretical lens. Findings will be reviewed with the IKT team and the Steering Committee.

*Analysis (feasibility of use)*

To understand the feasibility of the SDM strategy use, collected data from researcher reflective notes will be entered into an Excel database and analyzed descriptively by the researcher. Post-interaction interviews with CSW’s, community members, and managers will be analyzed descriptively with a six-step thematic analysis [46] process to determine feasibility for the use of the SDM strategy. Data will be contextualized using a postcolonial theoretical lens. Findings will be reviewed with the IKT team and the Steering Committee.

#### Research questions 8, 9 (stage e)

How can integrated knowledge translation research be conducted with FNIM communities?

What are the perceived determinants (that is, barriers and facilitators) for implementation of the peer supported SDM strategy?

*Design*

The use of the SDM strategy will be evaluated using consensus methods to determine contributions to the science of KT and to understand i) how integrated KT can be conducted with FNIM communities and ii) the perceived determinants for future implementation of the SDM strategy.

*Participant and procedures*

The Steering Committee and participating FNIM community members will be engaged to provide feedback to understand how integrated KT can be conducted with FNIM communities. An invitation to the community to meet will be made by poster/email and/or meeting with community leaders. The Steering Committee will be invited to review and comment by email or through an online or telephone meeting. Study documentation including meeting notes and a reflective journal that will have been kept by researcher(s) will be used in a process of document analysis. To identify the determinants for implementation of the strategy, an online and/or in-person presentation of study findings will be made to the Steering Committee and community members. Then, a survey and/or meeting discussion will be facilitated to obtain feedback about key facilitators and barriers to the use of the SDM strategy. Determinants for the future implementation of the SDM strategy will be developed and shared with the Steering Committee and the IKT team, and with final findings agreed upon by all. Final results will be disseminated using a plan agreed upon by the IKT team, Steering Committee members and participating communities.

*Analysis*

Study documentation (meeting notes, reflective journal, study paperwork) will undergo a process of document analysis and findings summarized and situated in relation to the current integrated KT literature. The information will then be reflected to the Steering Committee and participating communities in the form of an online survey and/or in-person presentations and that considers issues of sustainability [47]. Information will be collected and organized to describe stakeholder perspectives on needs, supports and barriers for the determinants of SDM strategy implementation.

### Limitations and strengths

Limitations of the study include failing to fully comprehend experiences of cancer care systems users as a small group of research participants are engaged from a limited number of communities. While the work proposed in this protocol may have some impacts it does not purport to directly tackle the substantial issue of institutional racism and exclusionary protocols and policies of cancer care and broader health systems. The strengths are that research approaches that strive for respectful and inclusive approaches towards FNIM people are being used. A Steering Committee and community participants are to be sought from distinct communities and that are anticipated to include one Inuit community, so that the socio-cultural context of potential users is incorporated into the tailoring and testing of the SDM strategy. As well, it is not the intent of the study to generate information that is generalizable to other communities, but to gather information that may potentially be used to collaboratively develop a larger study that can be designed to include a broader range of users designated as representative of groups for which the SDM strategy would be most important. To facilitate the evaluation of the data synthesis and credibility processes and demonstrate an awareness of how the study findings may potentially be transferred rich description, journal keeping, an audit trail, identification of clear outcomes, and contextualization of study findings are proposed.

## Discussion

There are few studies of interventions to influence the health status of FNIM populations within cancer care. This protocol outlines a plan to tailor and to field-test a previously developed SDM strategy for use and potential to enhance participation of FNIM people making cancer treatment decisions. In doing this it is anticipated that knowledge will be developed and that may be used to inform a subsequent effectiveness study, and to contribute to KT science.

An integrated KT approach in research is more likely to result in the co-production, exchange, and dissemination of knowledge [48] and with significant contributions anticipated. Indeed, this protocol has been structured to strengthen the work already underway with Cancer Care Ontario’s Aboriginal Cancer Control Unit [49] to further advance the achievements to promote a culturally safe space for dialogue between cancer clients and their healthcare providers throughout the cancer journey.

The anticipated outputs include descriptive papers and presentations about the research study’s integrated KT processes. Specifically, information is anticipated to be about the use of SDM in cancer care in ways that are ethical, equitable and upholds viable partnerships between researchers and knowledge users to advance development of peer support SDM strategy as a cancer care system intervention. The evidence built in this research study will be used to support the development of a proposal to conduct a multi-site implementation trial of peer-supported SDM.

Finally, integrated KT is not yet well defined in the research literature [50] and this research study proposes to define, use and describe integrated KT theory, tools and approaches. It is anticipated that this research study could provide an example for other cancer care initiatives that seek to engage with knowledge users and develop timely, applicable and acceptable cancer care.

## Abbreviations

CSW: Community support worker
FNIM: First Nations, Inuit, and Métis
KT: Knowledge translation
SDM: Shared decision making

## Footnotes

1 A lack of ethnic identifiers in Canadian administrative databases limits our ability to measure cancer burden in First Nations, Inuit and Metis populations. In Ontario, through appropriate data governance and sharing agreements, the Indian Register has been linked with the Ontario Cancer Registry to produce a report on cancer in First Nations people [51]. Currently, no comparable set of data/agreements exist with Metis or Inuit populations in Ontario.

2 Should other anticipated languages be preferred by participating community members for any part of the research process (Ojibway, Cree, Mohawk) they will be accommodated.
